# Prevalence and Factors of the Performed or Scheduled COVID-19 Vaccination in a Chinese Adult General Population in Hong Kong

**DOI:** 10.3390/vaccines9080847

**Published:** 2021-08-02

**Authors:** Yanqiu Yu, Mason M. C. Lau, Hui Jiang, Joseph T. F. Lau

**Affiliations:** Centre for Health Behaviours Research, JC School of Public Health and Primary Care, The Chinese University of Hong Kong, Hong Kong, China; yuyanqiu@link.cuhk.edu.hk (Y.Y.); mason-lau@cuhk.edu.hk (M.M.C.L.); 1155102419@link.cuhk.edu.hk (H.J.)

**Keywords:** COVID-19 vaccination, general population, China, perceptions, personal situation, trust toward the government

## Abstract

Background: Globally, COVID-19 vaccination programs have been rolled out. To inform health promotion, this study investigated the prevalence and associated factors of performance or being scheduled to perform at least one dose of COVID-19 vaccination (PSCV) in a Chinese adult general population. Methods: An anonymous, random telephone survey interviewed 500 adults aged 18–75 in Hong Kong, China from 14–27 May 2021. Results: The prevalence of PSCV was 21.0%, which was significantly lower among females and those aged ≤30. Positively associated factors of PSCV included perceived protection effect of vaccination, self-perceived physical fitness for vaccination, compulsory COVID-19 testing experience, perceived need to travel, general trust toward the government, and trust toward the governmental vaccination program, while negatively associated factors included perceived low efficacy of vaccination, concerns about side effects, and chronic disease status. Furthermore, the association between sex and PSCV was fully mediated by stronger concern about side effects and lower levels of self-perceived physical fitness for COVID-19 vaccination among females than males. Conclusion: Moderately low prevalence of COVID-19 vaccination was observed in Hong Kong, where there was no shortage of vaccine supply. To achieve herd immunity, health promotion is greatly warranted and may incorporate messages based on the findings of this study.

## 1. Introduction

Despite the extensive use of confinement strategies (e.g., lockdowns and social distancing), coronavirus disease 2019 (COVID-19) is still threatening many countries seriously and has accumulated over 182 million cases and 3.9 million deaths worldwide as of 2 July 2021 [1]. Vaccination has been seen by many as the ultimate solution to end the pandemic. With expedited developments, six COVID-19 vaccines have currently been approved for emergent use by the World Health Organization (WHO) since 31 December 2020 [2]. However, vaccine hesitancy remains a pivotal global concern. Prior to the approval of the COVID-19 vaccines, it ranged from 11.4% to 83.0% across countries [3,4,5,6,7,8], and was high in some countries (e.g., Arabs: 83.0% [7]; Malta: 48.2% [3]) while low in others (e.g., China: 11.4% [6]; Brazil: 14.7% [6]). Commonly reported factors of COVID-19 vaccine hesitancy included perceived efficacy and concerns about safety [4,9]. During the pre-marketing phase in September 2020, the prevalence of behavioral intention toward the COVID-19 vaccination (within the first six months of the vaccine’s availability) in the Hong Kong adult general population was only 24% given the COVID-19 vaccines having 80% efficacy and common mild side effects, which even dropped to 8.9% if the efficacy was 50% and mild side effects were common [10].

To control the COVID-19 pandemic, many countries rolled out free COVID-19 vaccination programs shortly after the approval of the first COVID-19 vaccine for emergency use [11]. As of 2 July 2021, over 3.1 billion doses of COVID-19 vaccines have been administered, covering 20.2% of the global population [11]. However, about half have been taken up in the U.S. and mainland China (0.3 and 1.2 billion doses, respectively) [11]. Except for a few countries (e.g., Israel: 57.1%; U.K.: 49.5%), most of the countries need to accelerate their vaccination rate to achieve herd immunity, including some developed countries that have suboptimal coverages (e.g., Canada: 19.5%; France: 35.9%; Italy: 31.2%) [11]. Except for China (44.5%), vaccination rates in Asian countries were in general low (e.g., Japan: 12.1%; Indonesia: 5.1%) [11], while they were only about 1–2% in some African countries, such as Uganda and Kenya [11]. Shortage of supply may be a major reason for low vaccination rates in most of the developing countries [12] and some developed countries (e.g., Canada and Australia) [13].

The impact of vaccine hesitancy is clearly demonstrated by the relatively low vaccination rates of some regions where free vaccines are fully available. To promote COVID-19 vaccination, it is warranted to understand factors of actual vaccination in the general population of such countries. The case of Hong Kong is illustrative. The Hong Kong government has received about 5.7 million doses of COVID-19 vaccines (about 1.2 million doses were returned because of package problems) for her population of 7.5 million; some more have been ordered [14]. As of 2 July 2021, the first-dose vaccination rate of Hong Kong residents aged ≥18 years, which was inclusive of ‘required’ vaccination for around 0.4 million foreign domestic workers, was 33.5% (2.3 million) [14]. The policy and environment in Hong Kong have been strongly facilitating vaccination. Currently, completely free choices between two types of free vaccines (i.e., Pfizer-BioNTech-Fosun with 95% efficacy in Phase III clinical trial and Sinovac Biotech with 50.3% efficacy in Phase III clinical trial) have been provided to all Hong Kong adult residents. The vaccination scheme started on 26 February 2021 (and for ages ≥12 on 3 June 2021). To facilitate vaccination, online booking is available at 29 conveniently located centers territory-wide; same-day appointments have usually been available. Incentives including relaxation of social distancing rules (e.g., size of table at restaurants and use of venues), vaccination leaves, and ad hoc monetary incentive (e.g., lucky draw for cash prize of 16,000 USD and apartments of 15 million USD) have also been offered to vaccinated people. There has been no vaccine shortage, but the vaccination rate has been moderate.

There is a dearth of studies investigating actual COVID-19 vaccination in the general population. Only two published studies have investigated the prevalence and factors of the actual uptake of COVID-19 vaccination in the U.S., but neither were conducted in the general population. One study reported 79% prevalence of COVID-19 vaccination behavior among healthcare workers, which was positively associated with educational level and perceived vulnerability to COVID-19 infection [15]. The other study reported that 49.8% of the inflammatory bowel disease patients in a Veterans Affairs cohort had received at least one dose of COVID-19 vaccination [16]. Some knowledge gaps about COVID-19 vaccination in the general population, therefore, exist. In literature, perceived efficacy versus perceived safety of COVID-19 vaccines are two opposing determinants of COVID-19 vaccination intention [17,18]. Most, if not all, of the vaccine hesitancy studies were conducted prior to the dissemination of the Phase III clinical trial results, and post-marketing data regarding effectiveness and safety were lacking. Indeed, only a few studies specified the levels of efficacy and safety when asking the questions on vaccination intention [10,19]. With more available information about the vaccines’ effectiveness and side effects in real-life settings, research on COVID-19 vaccination behavior is warranted, as the prevalence and factors of performance of vaccination may differ from those with the behavioral intention to receive the COVID-19 vaccination.

Individual experiences related to COVID-19 may affect COVID-19 vaccination. As mortality and severity of harm due to COVID-19 were higher among those with chronic diseases [20], those with chronic diseases may be motivated to take up COVID-19 vaccination. Such an association was indeed reported in previous studies on COVID-19 vaccination intention [10,21], although mixed findings have also been reported [22]. In parallel, people with chronic diseases might worry that their chronic disease status would elevate their vulnerability to severe side effects of COVID-19 vaccination, as many severe adverse events involved older people and those with chronic diseases [14]. It is warranted to understand the potential role of chronic disease status on COVID-19 vaccination in the general population. On a related note, those who subjectively believed that their health condition was not fit for COVID-19 vaccination were more likely to develop vaccine hesitancy. It is contended that self-rated physical fitness for vaccination would be positively associated with COVID-19 vaccination.

Compulsory massive COVID-19 testing for travelers and those having potential close contacts with infected cases (including those who live or work in proximity) have been widely implemented in Hong Kong [23]. Such an experience might change perceptions (e.g., perceived risk) related to COVID-19 vaccination and is a potential factor of COVID-19 vaccination. The need to travel is another potential individual-level factor considered in this study. International traveling has largely been suspended due to the COVID-19 pandemic and many people are eager to travel. There are recent discussions about ‘vaccine passports’ to facilitate vaccinated international travelers [24], which have been implemented in some countries.

Furthermore, the socio-ecological model postulates that individual-level (e.g., perceptions and personal experiences) and structural (e.g., cultural and political) factors are both key determinants of health behaviors [25]. The structural factors of trust toward the government in general and specific to the governmental vaccination program are part of social capital [26]. In literature, social capital was associated with health-related preventive behaviors, including influenza vaccination [27]. Consistently, trust toward the government was significantly associated with COVID-19 vaccination intention in multiple regions (e.g., Hong Kong [10] and some European countries [28,29]). It is particularly important in regions where trust toward the government is lacking or declining; Hong Kong is such a place because of the social movement and political conflicts occurring in the past few years [30]. However, associations between trust toward the government and COVID-19 vaccination behavior have not been tested.

Previous studies consistently showed that females possessed higher levels of vaccine hesitancy than males, including both COVID-19 vaccination [17,31,32] and influenza vaccination [33]. This is interesting as females tended to have higher levels of service utilization rates. For instance, female veterans with post-traumatic stress disorder were more likely than their male counterparts to utilize mental health, primary care, and emergency care services [34]. No empirical study has looked at potential reasons (mediators) explaining the sex difference in COVID-19 vaccination intention/behavior. Such information allows for tailored health promotion for the two sexes and was inspected in this study.

Given the background, the present study investigated the prevalence of performed or scheduled COVID-19 vaccination (PSCV), which is defined as having actually received at least one dose of COVID-19 vaccination or made an appointment to receive the first dose of COVID-19 vaccination, in the general adult population aged 18–75 years in Hong Kong. The present study also tested three types of potential factors of PSCV, including: (a) perceptions regarding COVID-19 vaccination (perceived efficacy and concerns about side effects), (b) personal situations (chronic disease status, self-perceived physical fitness for COVID-19 vaccination, experience in compulsory COVID-19 testing, and perceived need to travel), and (c) trust toward the government in general and trust toward the governmental vaccination program, which are two structural factors related to social capital. It is hypothesized that perceived efficacy, compulsory COVID-19 testing experience, self-perceived physical fitness for COVID-19 vaccination, and perceived need to travel would be positively associated with PSCV, while concerns about side effects would show a negative association. Without prior knowledge, we tested a two-sided alternate hypothesis whether chronic disease status would be positively or negatively associated with PSCV. A mediation hypothesis was tested, that any observed sex difference in PSCV, if it exists, would be mediated by the three aforementioned types of potential factors of PSCV. The findings would facilitate health promotion to increase COVID-19 vaccination.

## 2. Methods

### 2.1. Participants and Data Collection

Using a random telephone survey, a cross-sectional, population-based study was conducted among Chinese people aged 18–75 years in Hong Kong, China during 14–27 May 2021 and between 5–10 pm to avoid over-sampling non-working individuals. Telephone numbers were randomly drawn from the most updated residential telephone directory; about 85% of the Hong Kong households have landline phones. Unanswered telephone calls were given at least three attempts. The eligible household member whose birthday was closest to the survey date was interviewed. Appointments were made if necessary. No incentives were given to the participants. Participants could quit any time. The response rate, defined as the number of completed interviews (500) divided by the number of eligible contacts (880), was 56.8%.

Ethics approval was obtained from the Survey and Behavioral Research Ethics Committee of the Chinese University of Hong Kong (Ref No. SBRE-20-722). All participants were informed by trained interviewers about the content and objectives of the study. They were assured that participation was voluntary and rejection would have no negative consequences. Verbal informed consent was sought from all the participants; the interviewers were required to sign a form pledging they completed the required consent procedures.

### 2.2. Measures

#### 2.2.1. Background Variables

Background information was collected, including sex, age, educational attainment, marital status, and history of influenza vaccination.

#### 2.2.2. Performed or Scheduled COVID-19 Vaccination (PSCV)

The item assessed whether the participants had actually ever received at least one dose of COVID-19 vaccination or made an appointment to receive the first dose of COVID-19 vaccination (yes/no). This is the dependent variable of this study.

#### 2.2.3. Perceived Efficacy of COVID-19 Vaccination

Three items assessed perceptions related to the efficacy of COVID-19 vaccines (1 = strongly disagree to 5 = strongly agree), including (1) “COVID-19 vaccination could effectively protect yourself from contracting COVID-19” (protecting oneself), (2) “Due to insufficient efficacy, you would contract COVID-19 even if you had taken up COVID-19 vaccination” (low efficacy in general), and (3) “Currently available COVID-19 vaccines could not effectively protect you from contracting the mutated variants of COVID-19 virus (e.g., the Delta variant)” (low efficacy for preventing mutated variants of virus). The three items were used as separate independent variables as their composite summative scale (with the responses to the second and third items being reversed) showed an unacceptable low Cronbach’s alpha of 0.3.

#### 2.2.4. Concerns about Side Effects of COVID-19 Vaccination

Three items assessed concerns about side effects of the COVID-19 vaccines: (1) “COVID-19 vaccination may induce severe side effects and deaths (1 = strongly disagree to 5 = strongly agree)”, (2) “How much do you worry about COVID-19 vaccines’ side effects (1 = extremely low to 5 = extremely high)?”, and (3) “To what extent the current probability of severe side effects due to COVID-19 vaccination is acceptable to you? (1 = totally acceptable to 5 = totally unacceptable)”. The Cronbach’s alpha of the summative scale used in this study was 0.8.

#### 2.2.5. Personal Situations

Four items assessed some personal situations: (1) whether the participants had any one of the listed chronic diseases (e.g., diabetes, hypertension, chronic pulmonary diseases, myocardial infarction, cardiac failure, cerebrovascular diseases, ulcerative diseases, hepatic diseases, and tumor) (yes/no), (2) whether the participants self-perceived physical fitness for COVID-19 vaccination (1 = strongly disagree to 5 = strongly agree), (3) experience in compulsory COVID-19 testing (yes/no), and (4) level of perceived need to travel due to work, family visit, and tourism (1 = extremely low to 5 = extremely high).

#### 2.2.6. Trust toward the Government

Two items assessed trust toward the governmental vaccination program and toward the Hong Kong government in general (1 = strong mistrust to 5 = strong trust).

### 2.3. Statistical Analysis

The Software Pass 11.0 was used for sample size planning for logistic regression analysis involving continuous independent variables. Assuming that 10% to 30% of the individuals having the level of the independent variable of concern equal to the mean value have had PSCV, the planned sample size of 500 would yield the smallest detectable odds ratio (OR) in the range of 1.31 to 1.51 (power of 0.80 and alpha of 0.05, two-tailed) when these individuals were compared with those with value of the independent variable equal to mean plus one standard deviation. The sample size is, thus, adequate.

ANCOVA was used to test the sex differences in the continuous independent variables, adjusted for background factors; partial eta squared values were used to reflect the effect size. Univariable logistic regression analysis was conducted to test the associations between the studied background factors and PSCV. Multivariable logistic regression analyses were then conducted to test the individual associations between each of the independent variables and PSCV, adjusted for all the studied background factors. Crude odds ratio (ORc), adjusted odds ratio (ORa), and their respective 95% confidence intervals (95% CIs) were derived. The Baron and Kenny method [35] was used to test the mediations between sex and PSCV, which requires sex to be significantly associated with the studied factors (i.e., potential mediators) and the dependent variable. Logistic regression models that included both sex and one of the potential mediating variables, adjusting for the background variables, were fit for each of the potential mediators. The statistical significance of sex would become non-significant in the case of full mediations, or the ORa would diminish substantially while remaining statistically significant after adjusting for a potential mediator in the case of a partial mediation [35]. The analyses were performed by using SPSS 23.0. Statistical significance was defined as two-tailed *p* value < 0.05.

## 3. Results

### 3.1. Descriptive Statistics

Of the participants, over half were females (60.6%), aged 18–50 years old (56.0%), and being currently married (65.8%); 27.8% had attended colleges or universities (27.8%). About one-fourth self-reported having ever received the influenza vaccination (25.4%), currently having at least one type of chronic diseases (26.0%), and having experienced compulsory COVID-19 testing (22.4%). (see Table 1).

The mean scores (range) of the independent variables were described in Table 2. They included: (1) perceived efficacy of protecting oneself [3.5; (1–5)], (2) perceived low efficacy of COVID-19 vaccination in general [3.2 (1–5)], (3) perceived low efficacy of vaccination for preventing the mutated variants of COVID-19 virus [3.3 (1–5)], (4) concerns about side effects of COVID-19 vaccination [10.8 (3–15)], (5) self-perceived physical fitness for COVID-19 vaccination [3.3 (1–5)], (6) perceived need to travel [2.6 (1–5)], (7) trust toward the governmental vaccination program [2.8 (1–5)], and (8) trust toward the government in general [2.9 (1–5)].

### 3.2. Prevalence of PSCV

Of all the participants, 16.2% had completed at least one dose of COVID-19 vaccination; 4.8% had made an appointment for the first dose of vaccination. The prevalence of PSCV was hence 21.0%, which was 25.9% and 17.8% for males and females, respectively (*p* = 0.033, chi-square test), and 10.0%, 23.5%, and 22.7% for the 18–30, 31–50, >50 age groups, respectively (*p* = 0.030, chi-square test). Furthermore, 12.4% had completed two doses of COVID-19 vaccination.

### 3.3. Background Factors of PSCV

The results are summarized in Table 3. Background factors that were significantly associated with PSCV included: (1) male sex (ORc = 1.61, 95% CI: 1.04–2.49), (2) age groups of 31–50 years (reference = 18–30; ORc = 2.77, 95% CI: 1.24–6.15) and 51–75 years (reference = 18–30; ORc = 2.65, 95% CI: 1.20–5.87), and (3) history of influenza vaccination (ORc = 1.65, 95% CI: 1.03–2.63). Education level and marital status were not significant factors of PSCV.

### 3.4. Associations between Potential Factors and PSCV Adjusted for Background Variables

In Table 4, adjusted for all the studied background factors, factors that were significantly associated with PSCV included: (1) ***perceptions:*** the three efficacy factors of *(a) protecting oneself* (ORa = 2.63, 95% CI: 1.95–3.54), *(b) low efficacy in general* (ORa = 0.69, 95% CI: 0.54-0.90), *and (c) low efficacy of preventing mutated variants of virus* (ORa = 0.38, 95% CI: 0.26–0.56), and *concerns about side effects of COVID-19 vaccination* (ORa = 0.47, 95% CI: 0.40–0.55); (2) ***personal situation factors:*** *(a) chronic disease status* (ORa = 0.52, 95% CI: 0.28–0.97), *(b) self-perceived physical fitness for COVID-19 vaccination* (ORa = 2.69, 95% CI: 2.08–3.47), (*c) experience in compulsory COVID-19 testing* (ORa = 1.88, 95% CI: 1.14–3.11), *and (d) perceived need to travel* (ORa = 1.66, 95% CI: 1.30–2.12); and (3) ***trust toward the government:*** *(a) trust* *toward the government in general* (ORa = 2.26, 95% CI: 1.63–3.14) *and (b) trust toward the governmental vaccination program* (ORa = 3.60, 95% CI: 2.58–5.01).

### 3.5. Mediations between Sex and PSCV

Comparing the sex differences in the levels of independent variables, females were more likely than males to have concerns about side effects of COVID-19 vaccination (partial eta squared = 0.022; *p* < 0.001) and less likely to perceive physical fitness for COVID-19 vaccination (partial eta squared = 0.021; *p* < 0.001). There was no sex difference in the other independent variables (perceived efficacy, other personal situations, and trust toward the government). The results are presented in Table S1 in supplementary materials. The results of the mediation analysis were shown in Table S2 in supplementary materials. Adjusted for the studied background variables, the association between sex and PSCV became statistically non-significant after further controlling for concerns about side effects of vaccination (ORa = 1.42, 95% CI: 0.81–2.48) and self-perceived physical fitness for COVID-19 vaccination (ORa = 1.47, 95% CI: 0.88–2.44), respectively. Thus, the association between sex and PSCV was fully mediated by these two variables.

## 4. Discussion

This study found moderate prevalence of PSCV of 21% in the Hong Kong general adult population about three months since initiation of the vaccination program in Hong Kong, which was quite comparable to the 19.1% reported by the Hong Kong government as of 27 May 2021 (i.e., the last day of the survey) [14]. To attain herd immunity, the vaccination rate certainly needs to be improved. All the studied independent variables were significantly associated with PSCV. The association between sex and PSCV was fully mediated by concerns about side effects of COVID-19 vaccination and self-perceived physical fitness for COVID-19 vaccination.

Corroborating previous studies of vaccination intention conducted in Hong Kong [10] and overseas [32], females showed significantly lower prevalence of PSCV than males. In literature, females usually tended to have higher prevalence of health-related service utilization (e.g., mental health services) [36,37]. However, they showed stronger vaccine hesitancy (including influenza vaccination and COVID-19 vaccination) than males [17,21], although mixed findings have been reported [6,38]. This study may be the first to explore why females tended to have lower COVID-19 vaccination rates than males. It identified full mediations via stronger concerns about side effects of COVID-19 vaccination and lower perceived physical fitness for COVID-19 vaccination among females than males. Previous studies showed that females tended to have stronger worries about side effects of COVID-19 vaccination than males [39]. Some reports indeed showed more frequent side effects (including very rare but severe reactions) among females than males in Norway and the U.S., possibly due to sex differences in biological reactions (e.g., stronger immune responses in females than males) [40]. The stronger concerns about side effects among females might have deterred some of them from receiving the COVID-19 vaccination. Furthermore, a study found that females reported poorer self-perceived health than males during the COVID-19 pandemic [41]; they might, thus, feel less physically fit for COVID-19 vaccination. Future studies are still warranted to confirm and explain the implicative ‘global’ sex difference in COVID-19 vaccination. Additional health promotion efforts regarding concerns about side effects and self-perceived physical fitness for COVID-19 vaccination are needed to target and motivate females.

Age groups are potentially heterogeneous regarding COVID-19 vaccination intention. The prevalence of PSCV was only 10.0% for the 18–30 group, compared to 23.5% for the 31–50 group and 22.7% for the 51–75 age groups. A previous local study conducted in September 2020 (the pre-marketing phase) similarly reported prevalence of COVID-19 vaccination intention of only 7.9% and 12.7% in the 18–35 age group given vaccines having common mild side effects and 50% and 80% efficacy, respectively, which was significantly lower than that of other older age groups [10]. Similar trends of much higher vaccination hesitancy among young adults than older adults were found overseas [6,10,42,43]. For instance, it was 79.7%, 37.5%, and 45.8% in the age groups of <35, 51–65, and >65 age groups in Italy, respectively [43]. In the U.S., it was about 40% among those aged 18–34 versus 22% in the ≥55 age group [44]. Further comparative studies using similar tools are required to confirm this important finding of high vaccination hesitancy among adults aged ≤30. A plausibility to explain the low vaccination intention among younger adults in Hong Kong is that they tended to show a low level of trust toward the government for political reasons [45], while trust was associated with both COVID-19 vaccination intention in previous local studies [10,42] (and with actual vaccination in the present study). In literature, the positive association between trust in government and COVID-19 vaccine acceptance was also reported in a survey conducted in19 countries [6], while young people tended to trust the government less [46]. Limited by the small size of the 18–30 age group, the hypothesis about trust could not be tested in the present study. Again, comparative studies are greatly warranted. Interestingly, the prevalence of COVID-19 vaccination among the 30–50 and 51–75 age groups was very similar (23.5% and 22.7%). It was expected that the latter would have a higher vaccination rate than the former, according to the previously reported positive associations between age and COVID-19 vaccination intention. For instance, a local survey reported prevalence of COVID-19 vaccination intention of 40–50% among people aged >65 years versus 20–30% among those aged 36–64 [10,42]. A sense of uncertainty about safety might exist among older people aged >60 when they find out that the initial Phase III clinical trials of the COVID-19 vaccines did not include people aged >60 [47]. In addition, the frequent reports of severe side effects and deaths among older people occurring shortly after COVID-19 vaccination [14] might have increased vaccination hesitancy among older adults. Future cross-country research is, thus, needed to look at specific factors of COVID-19 vaccination in different age groups to support tailored health promotion strategies.

Given the convenient accessibility and supportive incentives, the prevalence of PSCV of about 30% in Hong Kong was unsatisfactory and inadequate to attain herd immunity. Health promotion programs, especially those targeting women and young adults, are certainly needed and may incorporate messages based on the present study’s finding. The hypothesis that perceived efficacy/perceived side effects would be positively/negatively associated with PSCV was supported. Thus, corroborating many studies on COVID-19 vaccine hesitancy [4,9,10], such factors remain key considerations of actual vaccination decisions and should be considered in related health promotion.

It is implicative that participants with chronic diseases were less likely than others to have received COVID-19 vaccination. About 1/4 of the participants self-reported having at least one of the listed chronic disease conditions. Although chronic disease status and, hence, the stronger need for protection may motivate COVID-19 vaccination, the frequent reports of deaths among chronic disease patients occurring soon after COVID-19 vaccination [14] might demotivate COVID-19 vaccination. Furthermore, health messages about chronic diseases and COVID-19 vaccination might have been ambivalent. Health authorities, such as the WHO, advised people with stable chronic disease conditions (e.g., hypertension, diabetes, and asthma) to receive COVID-19 vaccines [2,14], while some warnings about COVID-19 vaccination have also been issued to people with chronic disease conditions. For instance, the Hong Kong government recommends people with severe allergic reactions to vaccines, uncontrolled severe chronic disease, and pregnant and lactating women not to take up or defer taking up COVID-19 vaccination [14]. However, distinctions between stable versus controlled, severe versus non-severe, and specific chronic disease conditions may not always be straightforward to laypeople, especially among less educated and/or older people. Due to the length of the questionnaire, it is a limitation that we did not ask details about the self-reported chronic disease status (e.g., type, severity, and whether the disease was under control). Effective evidence-based health communication programs should confirm the suitability of COVID-19 vaccination among most of the chronic disease patients. Exceptions should be explained clearly (e.g., the types and level of severity not suitable for vaccination). Testimonials given by vaccinated chronic disease patients are potentially useful. Relatedly, perceived physical fitness for COVID-19 vaccination is important and positively associated with vaccination status; some comprehensible checklists to confirm suitability may also be helpful.

Some other personal experiences were significant factors of PSCV. Experience in compulsory COVID-19 testing was associated with COVID-19 vaccination, possibly due to increased perceived risk of COVID-19 infection or exposure to health education during the process. Systematic on-site health promotion and appointments for COVID-19 vaccination may be given to the testers. Perceived need to travel was another significant factor of PSCV. Hong Kong is an international hub, and people have great needs to travel to mainland China and overseas for work, tourism, and family reunion purposes. The development of international “vaccine passports” and lifting the restriction of quarantine in mainland China among vaccinated people may boost the vaccination rate.

The structural factors of trust toward the government (both general and specific to the local vaccination programs) were both significantly associated with PSCV. The findings corroborate many other local and international studies [6,10]. Trust toward the vaccination program might be improved by constant and transparent communications with the public about the number of vaccinated people and side effects, and endorsements given by reputable members of the society. The significant association between trust toward the government and PSCV is potentially a reflection of the politicization of COVID-19 vaccination and preventive behaviors [48,49,50]. Recently, the Hong Kong public faces serious deteriorations in such trust [45]. The role of trust toward the government and possibly also social capital factors in promoting COVID-19 vaccination need to be addressed.

Despite the current suboptimal vaccination rate, the accumulated number of COVID-19 vaccinations in Hong Kong keeps increasing steadily and has not decelerated [51]. A previous vaccination intention study suggested that 68.2% of the population was adopting a wait-and-see attitude [10]. With more safety data and testimonials disseminated, it is expected that some of the late majority and laggards, according to the diffusion of innovation theory [52], would start vaccinating. The descriptive norm effect (knowing that many people having been vaccinated) of the theory of planned behaviors [53] and the observation learning effect of the social cognitive theory [54] both suggest an increase in the vaccination rate in the future. The final vaccination rate in Hong Kong is subject to unpredictable incidences, such as outbreaks of mutated variants of COVID-19 virus and related changes in individuals’ risk perception. Keeping in mind that this study was conducted in the early phase of COVID-19 vaccination (three months after COVID-19 vaccination started in Hong Kong), serial surveillance and international comparisons are warranted.

The study has several limitations. First, as COVID-19 vaccination is socially desirable, reporting bias may exist. Second, causal or temporal inferences could not be claimed due to the cross-sectional nature of this study. Third, although the response rate (56.8%) was comparable to other local telephone surveys, the responses between the participants and non-participants might differ, but comparisons were not feasible. Nonetheless, although females were slightly over-represented, the age distribution of this study was comparable to that of Hong Kong census data of 2019 in general. Fourth, participants aged >75 years were not included in this study. Fifth, some of the scales were constructed for this study as validated scales were unavailable. Finally, the study may have missed important factors such as self-efficacy in vaccination.

## 5. Conclusions

In conclusion, moderately low prevalence of COVID-19 vaccination behavior was observed in the adult general population in Hong Kong, where supply of vaccines was not an issue and plenty of measures have been adopted to facilitate COVID-19 vaccination. It is still uncertain whether Hong Kong will be able to achieve herd immunity via vaccination in the recent future, but certainly, strong health promotion efforts are required, and its tailored design may take into account the age/sex differences and the associations found in this study.

## Figures and Tables

**Table 1:** Descriptive statistics of the categorical background and independent variables (*n* = 500).

	N	%
**Background factors**		
Sex		
Female	303	60.6
Male	107	39.4
Age groups (years)		
18–30	80	16.0
31–50	200	40.0
51–75	220	44.0
Educational attainment		
≤Primary school	99	19.8
Middle school	254	50.8
≥College	139	27.8
Missing data	8	1.6
Marital status		
Married	329	65.8
Others (e.g., single and widowed)	168	33.6
Missing data	3	0.6
History of influenza vaccination		
No	373	74.6
Yes	127	25.4
**Personal situations**		
Chronic disease status		
No	370	74.0
Yes	130	26.0
Experience in compulsory COVID-19 testing		
No	388	77.6
Yes	112	22.4

**Table 2:** Descriptive statistics of the continuous independent variables (n = 500).

	Range	Mean	SD
**Perceived efficacy of COVID-19 vaccination**			
Protecting oneself	1–5	3.5	1.0
Low efficacy in general	1–5	3.2	0.9
Low efficacy for preventing mutated variants of virus	1–5	3.3	0.7
**Concerns about side effects of COVID-19 vaccination**	3–15	10.8	2.5
**Personal situations**			
Self-perceived physical fitness for COVID-19 vaccination	1–5	3.3	1.2
Perceived need to travel	1–5	2.6	1.2
**Trust toward the government**			
Trust toward the governmental vaccination program	1–5	2.8	1.0
Trust toward the government in general	1–5	2.9	0.9

Note. SD = standard deviation.

**Table 3:** Background factors of performed or scheduled COVID-19 vaccination (n = 500).

	Performed or Scheduled COVID-19 Vaccination (PSCV)	
	ORc (95% CI)	*p*-Value
Sex		
Female	Ref = 1.0	
Male	1.61 (1.04–2.49)	0.031
Age groups (years)		
18–30	Ref = 1.0	
31–50	2.77 (1.24–6.15)	0.013
51–75	2.65 (1.20–5.87)	0.016
Educational attainment		
≤Primary school	Ref = 1.0	
Middle school	1.30 (0.72–2.35)	0.380
≥College	1.24 (0.65–2.38)	0.520
Missing data	NA	
Marital status		
Married	Ref = 1.0	
Others (e.g., single and widowed)	0.74 (0.46–1.18)	0.203
Missing data	NA	
History of influenza vaccination		
No	Ref = 1.0	
Yes	1.65 (1.03–2.63)	0.037

Note. ORc = crude odds ratio; CI = confidence interval; Ref = reference group; NA = not applicable due to low frequencies.

**Table 4:** Adjusted associations between potential factors and performed or scheduled COVID-19 vaccination (n = 500).

	Performed or Scheduled COVID-19 Vaccination (PSCV)	
	ORa (95% CI)	*p*-Value
**Perceived efficacy of COVID-19 vaccination**		
Protecting oneself	2.63 (1.95–3.54)	<0.001
Low efficacy in general	0.69 (0.54–0.90)	0.005
Low efficacy for preventing mutated variants of virus	0.38 (0.26–0.56)	<0.001
**Concerns about side effects of COVID-19 vaccination**	0.47 (0.40–0.55)	<0.001
**Personal situations**		
Chronic disease status	0.52 (0.28–0.97)	0.039
Self-perceived physical fitness for COVID-19 vaccination	2.69 (2.08–3.47)	<0.001
Experience in compulsory COVID-19 testing	1.88 (1.14–3.11)	0.014
Perceived need to travel	1.66 (1.30–2.12)	<0.001
**Trust toward the government**		
Trust toward the governmental vaccination program	3.60 (2.58–5.01)	<0.001
Trust toward the government in general	2.26 (1.63–3.14)	<0.001

Note. ORa = adjusted odds ratio; CI = confidence interval. Adjusted associations were adjusted for sex, age groups, educational level, marital status, and history of influenza vaccination.

## Data Availability

The data was available on reasonable request.

## Supplementary Materials

The following are available online at https://www.mdpi.com/article/10.3390/vaccines9080847/s1, Table S1: Sex differences in the levels of potential factors of performed or scheduled COVID-19 vaccination, Table S2: Mediation analyses on the sex difference in the prevalence of performed or scheduled COVID-19 vaccination.
