# Supplementary material for: Prevalence and Factors of the Performed or Scheduled COVID-19 Vaccination in a Chinese Adult General Population in Hong Kong
Source: Vaccines (Basel). 2021 Aug 2;9(8):847. doi: 10.3390/vaccines9080847 (PMC8402378; doi:10.3390/vaccines9080847)
Supplement: Supplementary file 1 [file vaccines-09-00847-s001.zip › vaccines-1304797-supplementary.pdf]

Table S1. Sex differences in the levels of potential factors of performed or scheduled COVID-19 vaccination (n = 500).

	Males	Females	Effect	*p*-
	Mean, SD	Mean, SD	size	value
**Perceived efficacy of COVID-19 vaccination**				
Protecting oneself	3.4, 1.0	3.5, 0.9	0.001	0.927
Low efficacy in general	3.17, 0.9	3.18, 0.9	0.001	0.601
Low efficacy for preventing mutated variants of virus	3.3, 0.7	3.4, 0.7	0.001	0.437
**Concerns about side effects of COVID-19 vaccination**	10.4, 2.3	11.2, 2.6	0.022	0.001
**Personal situations**				
Chronic disease status ¶	20.8	29.4	0.71	0.204
Self-perceived physical fitness for COVID-19 vaccination	3.6, 1.2	3.1, 1.2	0.021	0.001
Experience in compulsory COVID-19 testing ¶	20.3	23.8	0.86	0.508
Perceived need to travel	2.6, 1.2	2.5, 1.2	0.001	0.643
**Trust toward the government**				
Trust toward the governmental vaccination program	2.87, 0.9	2.90, 0.9	0.001	0.426
Trust toward the government in general	2.7, 1.1	2.9, 1.0	0.001	0.693

Note. SD = Standard deviation. , Proportions were used to statistically describe these variables. Sex differences in chronic disease status and experience in compulsory COVID-19 testing were tested by using multivariable logistic regression analysis (with adjusted odds ratio being effect size), while sex differences in the other variables were tested by using ANCOVA (with partial eta squared being effect size). All the analyses were adjusted for age, educational level, marital status, and history of influenza vaccination.

Table S2. Mediation analyses on the sex difference in prevalence of performed or scheduled COVID-19 vaccination.

	Performed or scheduled COVID-19 vaccination (PSCV)		*p* of indirect effect	Mediation effect size
	ORa (95% CI)	*p*		
Model 1				
Sex (male)	1.77 (1.12-2.80)	0.015	-	-
Model 2				
Sex (male)	1.88 (1.16-3.04)	0.011	-	-
Protecting oneself	2.63 (1.95-3.54)	< 0.001	0.436	-
Model 3				
Sex (male)	1.75 (1.10-2.77)	0.018	-	-
Low efficacy in general	0.69 (0.54-0.90)	0.005	0.845	-
Model 4				
Sex (male)	1.77 (1.10-2.84)	0.018	-	-
Low efficacy for preventing mutated variants of virus	0.38 (0.26-0.56)	< 0.001	0.726	-
Model 5				
Sex (male)	1.42 (0.81-2.48)	0.218	-	-
Concerns about side effects of COVID-19 vaccination	0.47 (0.40-0.55)	< 0.001	0.001	Full mediation
Model 6				
Sex (male)	1.73 (1.09-2.75)	0.019	-	-
Chronic health status	0.52 (0.28-0.97)	0.039	0.279	-
Model 7				
Sex (male)	1.47 (0.88-2.44)	0.141	-	-
Self-perceived physical fitness for COVID-19 vaccination	2.69 (2.08-3.47)	< 0.001	0.001	Full mediation
Model 8				
Sex (male)	1.83 (1.15-2.90)	0.011	-	-
Experience of compulsory COVID-19 testing	1.88 (1.14-3.11)	0.014	0.522	-
Model 9				
Sex (male)	1.99 (1.12-3.53)	0.018	-	-
Perceived need to travel	1.66 (1.30-2.12)	< 0.001	0.478	-
Model 10				
Sex (male)	1.83 (1.11-3.02)	0.018	-	-
Trust toward the governmental vaccination program	3.60 (2.58-5.01)	< 0.001	0.675	-
Model 11				
Sex (male)	2.33 (1.29-4.22)	0.005	-	-
Trust toward the government in general	2.26 (1.63-3.14)	< 0.001	0.101	-

Note. ORa = Adjusted odds ratio. CI = Confidence interval. The models were adjusted for age, educational level, marital status, and history of influenza vaccination.
